# Secreted PD-L1 alleviates inflammatory arthritis in mice through local and systemic AAV gene therapy

**DOI:** 10.3389/fimmu.2025.1527858

**Published:** 2025-02-03

**Authors:** Wenjun Li, Junjiang Sun, Susi Feng, Ariana La Rosa, Panli Zhang, Eveline Y. Wu, Richard Loeser, Chengwen Li

**Affiliations:** ^1^ Gene Therapy Center, University of North Carolina at Chapel Hill, Chapel Hill, NC, United States; ^2^ Division of Oral and Craniofacial Biomedicine, University of North Carolina Adams School of Dentistry, Chapel Hill, NC, United States; ^3^ Department of Pediatrics, University of North Carolina at Chapel Hill, Chapel Hill, NC, United States; ^4^ Division of Rheumatology, Allergy, and Immunology, University of North Carolina, Chapel Hill, NC, United States; ^5^ Carolina Institute for Developmental Disabilities, University of North Carolina at Chapel Hill, Chapel Hill, NC, United States

**Keywords:** AAV, RA, intra-articular, systemic, soluble

## Abstract

**Introduction:**

Rheumatoid arthritis (RA) primarily affects the joints but can also affect multiple organs and profoundly impacts patients’ ability to carry out daily activities, mental health, and life expectancy. Current treatments for RA are limited in terms of duration, efficacy, and adverse effects. PD-L1 is a checkpoint protein that plays important roles in immune regulation and has been implicated in the initiation and progression of multiple autoimmune diseases.

**Method:**

In a previous study, we demonstrated that intra-articular injection with adeno-associated virus (AAV) vectors encoding wild type PD-L1 improved local inflammation in the joint in the collagen-induced arthritis (CIA) mouse model of RA. To further improve efficacy, we explored AAV-mediated delivery of the soluble PD-L1 (sPD-L1) to CIA mice.

**Result:**

After intra-articular injection of AAV6 vectors expressing the optimal isoform of sPD-L1 (shPD-L1), more potency was observed when compared to wild type PD-L1, with a lower dose of AAV6/shPD-L1 needed for arthritis improvement. To study the therapeutic effect of systemic expression of sPD-L1, we administered AAV8/shPD-L1 gene therapy in CIA mice via retro-orbital injection and found significant improvements in joint inflammation and paw swelling, exhibiting similar phenotypes to that in naïve mice. The levels of total immunoglobulin and anti-collagen specific antibodies were lower in AAV8/shPD-L1 treated CIA mice than those in controls. The levels of pro-inflammatory cytokines in blood were also significantly decreased in shPD-L1 treated mice. Additionally, T cell apoptosis rates in the spleen showed a 2-fold increase in treated mice. Finally, we investigated the therapeutic effect of AAV/shPD-L1 via intramuscular injection. After injection of AAV6/shPD-L1, decreased paw swelling, reduced joint inflammation, and lower levels of pro-inflammatory cytokines in blood were achieved. The therapeutic effect of shPD-L1 was dose dependent via intramuscular treatment with AAV vectors.

**Conclusion:**

In conclusion, the findings in this study suggest that intra-articular injection of AAV vectors encoding sPD-L1 results in greater therapeutic benefit on arthritis, and systemic AAV/sPD-L1 is able to block the development of inflammatory arthritis with inhibition of the systemic immune response, underlining the potential of gene therapy with systemic delivery of shPD-L1 via AAV vectors in RA.

## Introduction

Rheumatoid arthritis (RA) is a complex autoimmune disease primarily characterized by the accumulation of autoantibodies, cytokines, and immune cells into the joint synovium (1). Subsequent joint destruction can lead to decreased quality of life, disability, and early mortality. As RA affects approximately 1% of individuals worldwide, sustained efforts to advance RA treatment regimens are important for mitigating the considerable societal and economic burdens of disease.

One important category of drugs used in the treatment of RA is disease-modifying antirheumatic drugs (DMARDs) (2–5). Biological DMARDs (biologics) have emerged as a promising alternative to conventional synthetic DMARDs for the treatment of inflammatory arthritis such as rheumatoid and psoriatic arthritis. Biologics target specific inflammatory molecules or pathways (3), including interleukins, TNF-α (6), and B-cell and T-cell survival and activity (7, 8).

However, these treatments typically require repeat dosing and frequent administration and often result in unsatisfactory outcomes (9–11). Compared to protein therapy or traditional pharmaceutical drugs, gene therapy with adeno-associated virus (AAV) is a promising candidate for addressing the short duration of biologic protein-based therapies, as it can provide long-term expression of the packaged therapeutic genes after just a single dose of administration. In particular, the salient role of immune cell dysfunction in RA pathogenesis indicates the programmed cell death protein-1 (PD1)/programmed death-ligand 1 (PD-L1) axis is an attractive candidate for gene therapy given that the binding of PD-L1 to the PD1 receptor on lymphocytes results in immune cell suppression (12).

In a previous study, we used AAV-delivered wild type PD-L1 for local RA treatment, with expression being confined to the knee joint. Prophylactic and therapeutic intra-articular injections showed efficiency in preventing and blocking arthritis progression, respectively (13). However, the effects were limited, partly due to the relatively low number of transduced cells and the use of wild type PD-L1 with the transmembrane domain. Soluble proteins, such as cytokines, antibodies, and peptides, are commonly used for preventing or treating systemic diseases (14). Part of their appeal is that soluble proteins occupy a larger range of biomedical applications than insoluble proteins, with multiple approaches and routes having been explored (15). Gong, et al. demonstrated the strong affinities of soluble PD-L1 variants with PD1, with a small number being able to block the effects from PD-L1 targeting therapies – suggesting the potential physiological role of these isoforms as decoy targets (16). Similarly, Sagawa, et al. reported a different soluble splicing variant, PDL1–vInt4. Although no immunosuppressing function was detected, results suggested its role as a decoy for PD-L1, offering a potential mechanism for cancerous resistance to anti-PD-L1 treatment (17).

In this study, we cloned three soluble PD-L1(sPD-L1) variants by modifying sequences in the transmembrane domain and found the shPD-L1 variant displayed the most potent immunosuppressive effects. We then investigated shPD-L1 for AAV vector-mediated gene delivery via various administration routes. Our results showed that both local intra-articular and systemic expression of sPD-L1 could block the progression of arthritis in a collagen-induced arthritis (CIA) mouse model. The expression of sPD-L1 alleviated paw swelling and inflammation by decreasing pro-inflammatory cytokine production as well as regulating the levels of autoantibody production.

## Results

### Soluble PD-L1 showed higher transgene expression and efficiency compared to wild type PD-L1 in CIA mice treated with intra-articular injection of AAV vectors

We aimed to develop a soluble PD-L1 variant that might be more advantageous due to its ability to be secreted by any transduced cells in the joint and would be effective across a wider range of cell types compared to the wild type non-secretory PD-L1. Soluble variants of PD-L1 are isoforms of the ligand that are exported extracellularly, rather than being expressed on the cell surface like the wild type form, through processes such as extrusion into extracellular spaces and blood. Soluble PD-L1 can be generated either by proteolysis or alternative mRNA splicing (18), with variants reported in tumor patients (17). For instance, as reported in Gong, et al. (16), these splicing variants appear to have mutations in the transmembrane domain that allow for secretion.

**Figure 1:**
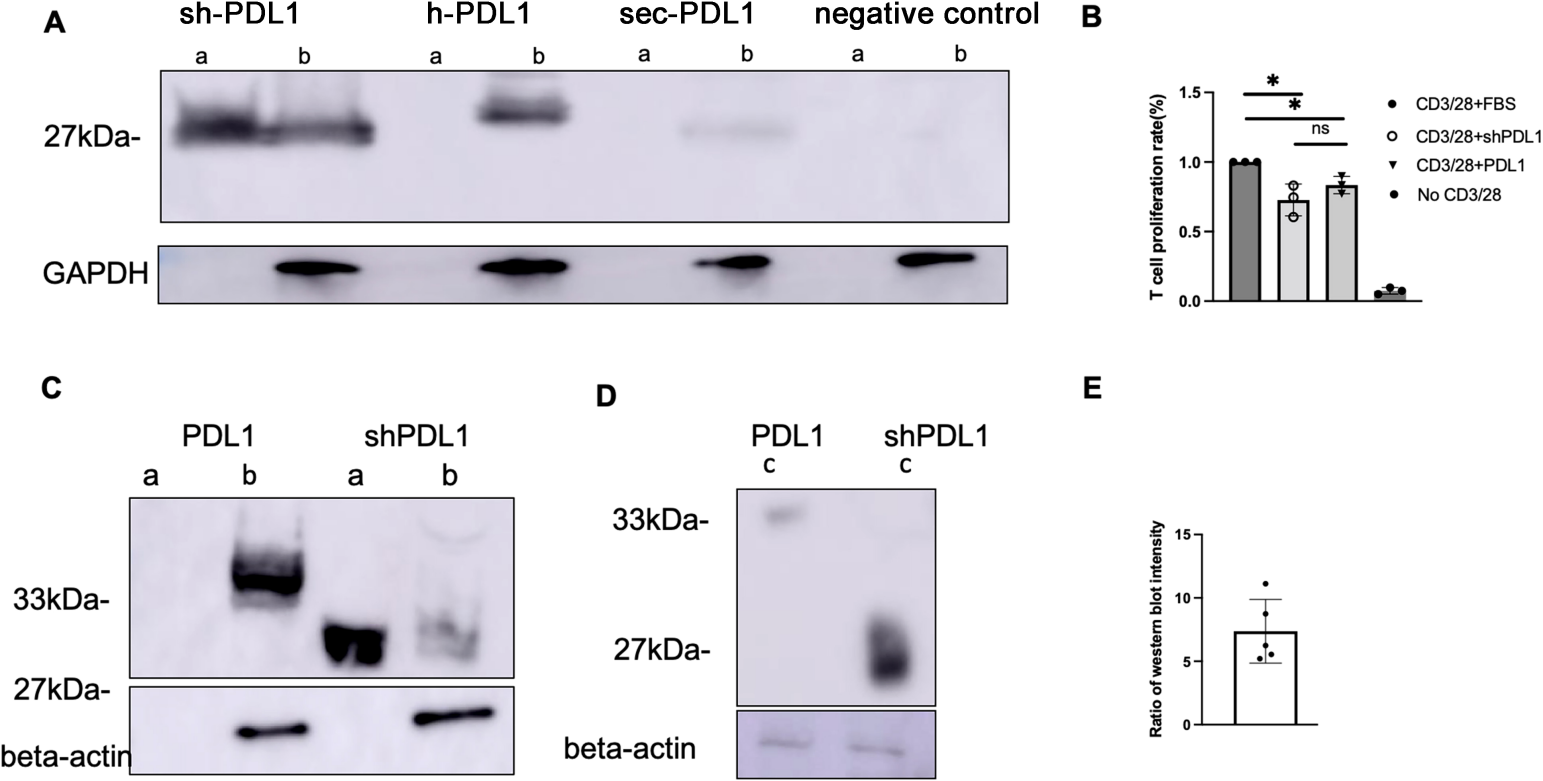
Detection of shPD-L1 secretion and functional activity *in vitro*. **(A)** Western blot analysis (n=3) for soluble PD‐L1 protein expression. 48h after transfection of pTR/CBh-shPD-L1, pTR/CBh-hPD-L1, pTR/CBh-secPD-L1or pTR/CBh-GFP into HEK-293 cell lines, the supernatant and cell lysate were collected for detection of soluble PD-L1 expression by western blot with antibodies against His tag. a, supernatant, b, cell lysate. **(B)** T cell proliferation rate. Purified splenic T cells were stained with CellTrace Violet dye, then co-cultured with FBS, positive control or shPD-L1 in the presence of anti-CD3/anti-CD28 for 72h. The proliferation of positively stained cells(n=3) was analyzed with flow cytometry. Data were analyzed using one-way ANOVA followed by Bonferroni multiple comparison test for group comparisons. *p < 0.05. **(C)** Western blot analysis for protein expression of shPD-L1 and wild type PD-L1. a, supernatant, b, cell lysate. **(D)** Western blot analysis for total protein expression in both supernatant and cell lysates of shPD-L1 and PD-L1. c, a total mixture of supernatant and cell lysate. **(E)** The ratio of total protein expression level (n=5) of shPD-L1 to wild type PD-L1.

In order to enhance transgene secretion, we synthesized and cloned three distinct PD-L1 variants with alterations in their transmembrane domain: shPD-L1, hPD-L1, and sec PD-L1 (**Supplementary Data**). Each variant incorporated a His-tag at the C-terminus for detection purposes. The three variant sequences were transfected into HEK-293 cells, and after 48 hours, expression of the three PD-L1 variants was analyzed using an anti-His-tag antibody. The shPD-L1 variant was successfully secreted as indicated by its expression in both the supernatant and cell lysates, compared to the other two variants, which were only detected in cell lysates (**Figure 1A**). To test the efficacy of shPD-L1, we conducted T cell functional assays by analyzing the effect on T cell proliferation *in vitro*. Incubation with shPD-L1 resulted in a significant decrease in T cell proliferation compared to incubation with FBS (**Figure 1B**). We also compared T cell proliferation in cells incubated with equal amounts of shPD-L1 and PD-L1 molecules and observed no significant difference between the two groups (**Figure 1B**). Moreover, compared to the wild type PD-L1, the total expression level of shPD-L1 in HEK-293 cells was observed to be approximately 7 times higher (**Figures 1C–E**).

**Figure 2:**
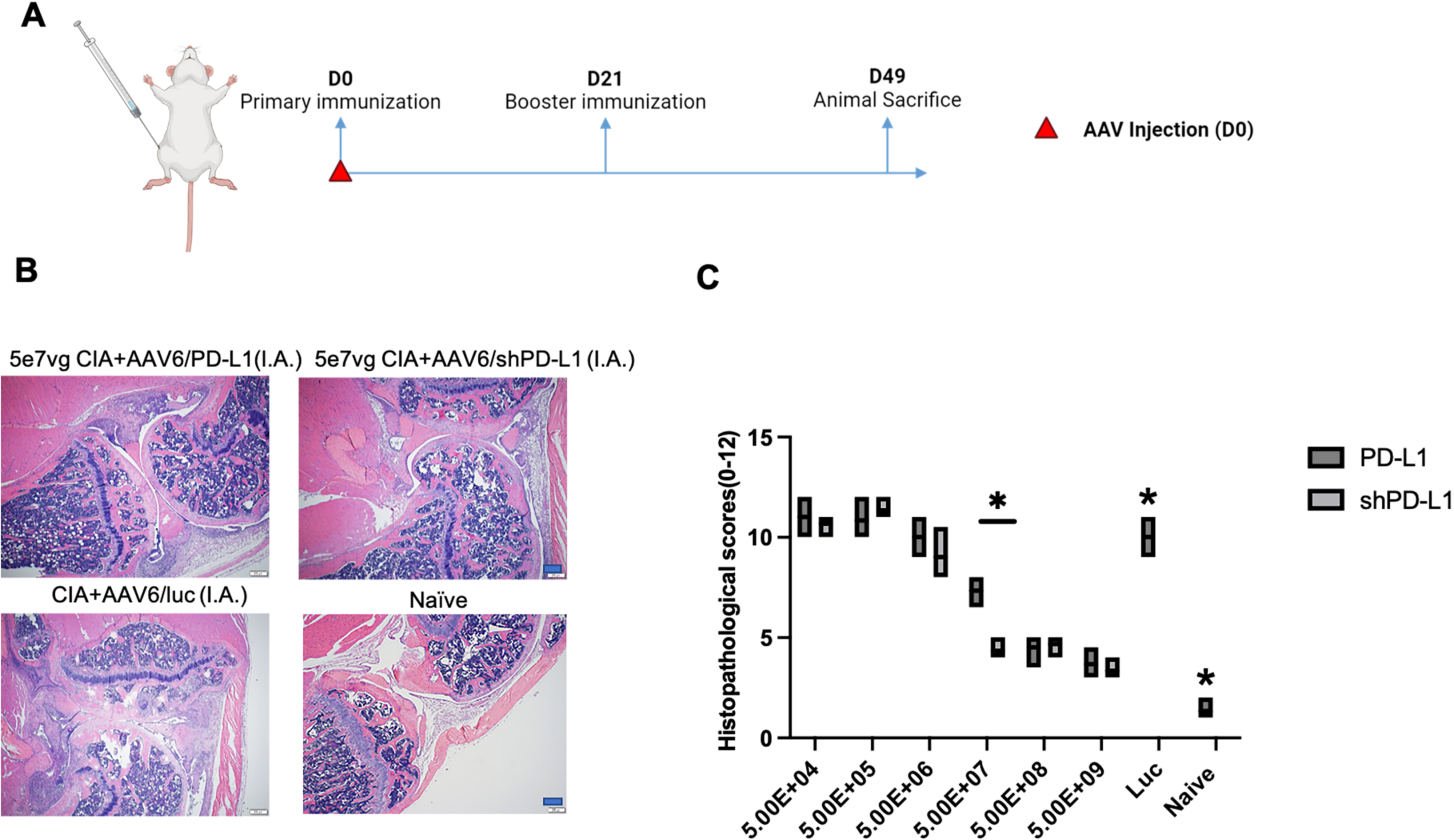
shPD-L1 showed a higher efficacy in alleviating joint inflammation than PD-L1 in CIA mice. **(A)** Diagram of mouse injections. Mice underwent primary immunization and intraarticular injection simultaneously on day 0. Booster injections were performed on day 21, and mice were sacrificed on day 49. **(B)** Joint histology analysis with H&E staining. Representative images of H&E staining from the CIA mouse knees intraarticularly injected with 5e7 vg of AAV6/shPD-L1, AAV6/PD-L1, AAV6/luc, or naïve mice at week 7 are shown (n=5, bar=200μm). **(C)** The knee joint histological score (n=5). Different doses of AAV6/shPD-L1 or AAV6/PD-L1 were injected into the knees of CIA mice on day 0. On the same day, the primary immunization with type II collagen was applied, and on day 21, the booster immunization was applied. At week 7, joints were collected for histology analysis. Histopathological evaluation was performed and scored by two independent observers for the following changes: synovial hyperplasia, leukocyte infiltration, pannus formation, and cartilage necrosis/erosion. Data are represented as means ± SEM. Data were analyzed using two-tailed unpaired Student’s t test. *p < 0.05.

Subsequently, shPD-L1 was packaged into AAV6-shPD-L1 for *in vivo* studies (**Figure 2A**). To compare the efficiency of shPD-L1 and PD-L1, we carried out a dose-response study via intra-articular injection in the CIA mouse model, using doses ranging from 5x10^9^ to 5x10^4^ vector genome (vg) with 10-fold serial dilutions, each group contained five mice. The results revealed that shPD-L1 maintained its therapeutic efficacy at a lower dose in comparison to wild type PD-L1. Specifically, AAV6-PD-L1 lost its ability to mitigate arthritis when the dose decreased to 5x10^7^ vg, whereas shPD-L1 continued to show efficacy (p <0.05) (**Figures 2B, C**). These findings underscored the enhanced potency of shPD-L1 over wild type PD-L1.

### Local shPD-L1 from intra-articular injection of AAV vectors did not impact the systemic immune response

To examine if shPD-L1 leaked into the blood stream post intra-articular AAV administration, we tested the shPD-L1 levels in serum using an anti-his tag ELISA kit and found that the shPD-L1 protein was not detected (data not shown). This is also consistent with the finding that overall paw swelling symptoms remain, with no significant difference compared to untreated CIA mice (**Supplementary Data**). We also analyzed the T cell composition in spleen cells by staining for CD4+ and CD8+ positive cells. No significant differences were observed between the groups (**Supplementary Data**). Further, we evaluated the impact on the systemic autoimmunity by examining the anti-collagen II antibody levels. Similar to the control treated CIA mice, the anti-collagen II antibody levels in serum were not altered by intra-articular shPD-L1 treatment (**Supplementary Data**).

### Improved joint inflammation and paw swelling of CIA mice intravenously treated with AAV8/shPD-L1

Given that PD-L1 is an immunoinhibitory protein, its systemic introduction into the context of RA could lead to two potential outcomes: it might alleviate symptoms on a systemic level, or it could introduce adverse effects, including immune dysfunction, among others. To assess the systemic impact on immunity of shPD-L1, we administered AAV8 vectors encoding shPD-L1 (AAV8/shPD-L1) or luciferase as a control (AAV8/luc) to CIA mice via intravenous injections. We choose AAV8 due to the superior liver-directed transduction efficiency of AAV8 relative to other AAV serotypes (19).

**Figure 3:**
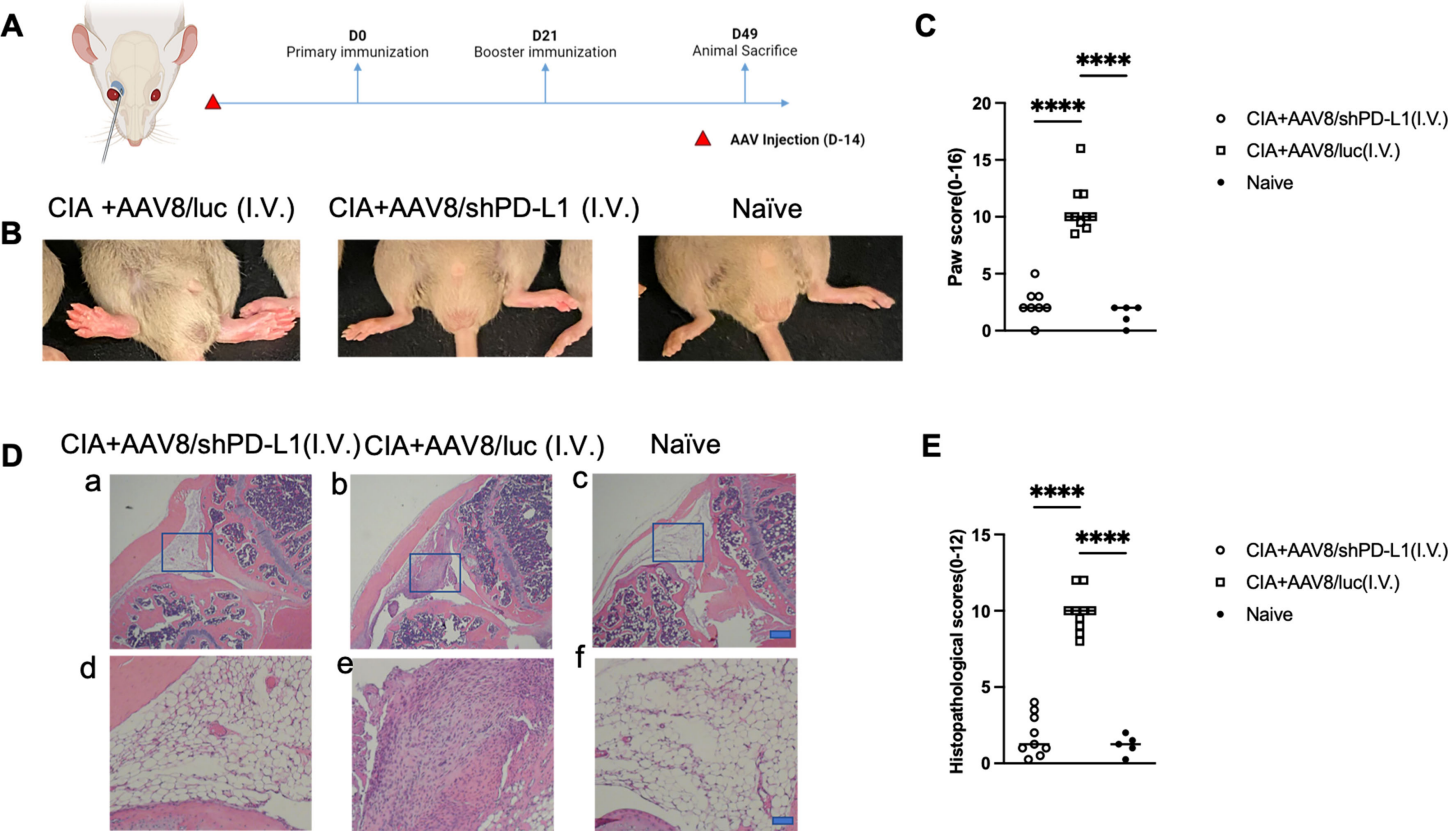
Joint histology and paw swelling of CIA mice intravenously treated with AAV8/shPD-L1. **(A)** Diagram of mouse injections. Mice were injected intravenously via the retro-orbital vein 2 weeks prior to primary immunization on day 0, and booster injections were performed on day 21. Sacrifice was performed on day 49. **(B)** The representative images of mice with swollen paws. The leftmost picture was CIA mice intravenously injected with AAV8/luc, the middle picture was CIA mouse intravenously injected with AAV8/shPD-L1, the rightmost picture was naïve mouse. **(C)** Paw score of mice treated with AAV8/shPD-L1(n=10), AAV8/luc(n=10), and naïve mice(n=5). The paw swelling score was assessed independently by two observers, with each of the four paws receiving a score ranging from 0 to 4. The total score for each mouse was calculated by summing the individual paw scores. Data were analyzed using one-way ANOVA followed by Bonferroni multiple comparison test for group comparisons.****, p < 0.001 **(D)** Joint histology analysis with H&E staining. Representative images of H&E staining from the CIA mouse knees intravenously injected with 2e11 vg of AAV8/shPD-L1, CIA+AAV8/luc, or naïve mice at week 7 are shown (a-c, bar=200μm; d-f, bar=20μm). **(E)** Histological scores of CIA mice intravenously injected with AAV8/shPD-L1 (n=10), AAV8/luc (n=10), and naïve mice (n=5). Data were analyzed using one-way ANOVA followed by Bonferroni multiple comparison test for group comparisons.****, p < 0.001.

First, we studied the clinical manifestation of RA in CIA mice. 2x10^11^vg of AAV8/shPD-L1 vectors were intravenously administered to the mice two weeks prior to CIA induction (**Figures 3A**). CIA mice treated with AAV8/shPD-L1 showed similar clinical scores, evaluated by visually assessing paw swelling, to naïve mice and exhibited around 4-fold less redness and paw swelling when compared to CIA AAV8/luc control mice (2.4 ± 1.4 vs 10.7 ± 2.2) (p <0.05) (**Figures 3B, C**). After 7w, mice were sacrificed, and the knee joints were collected and stained with hematoxylin and eosin (H&E). The results showed around 5-fold less inflammation (1.8 ± 1.4 vs 9.9 ± 1.3) (p <0.05) in the H&E stains from shPD-L1 treated mice (**Figures 3D, E**) compared to CIA control mice.

**Figure 4:**
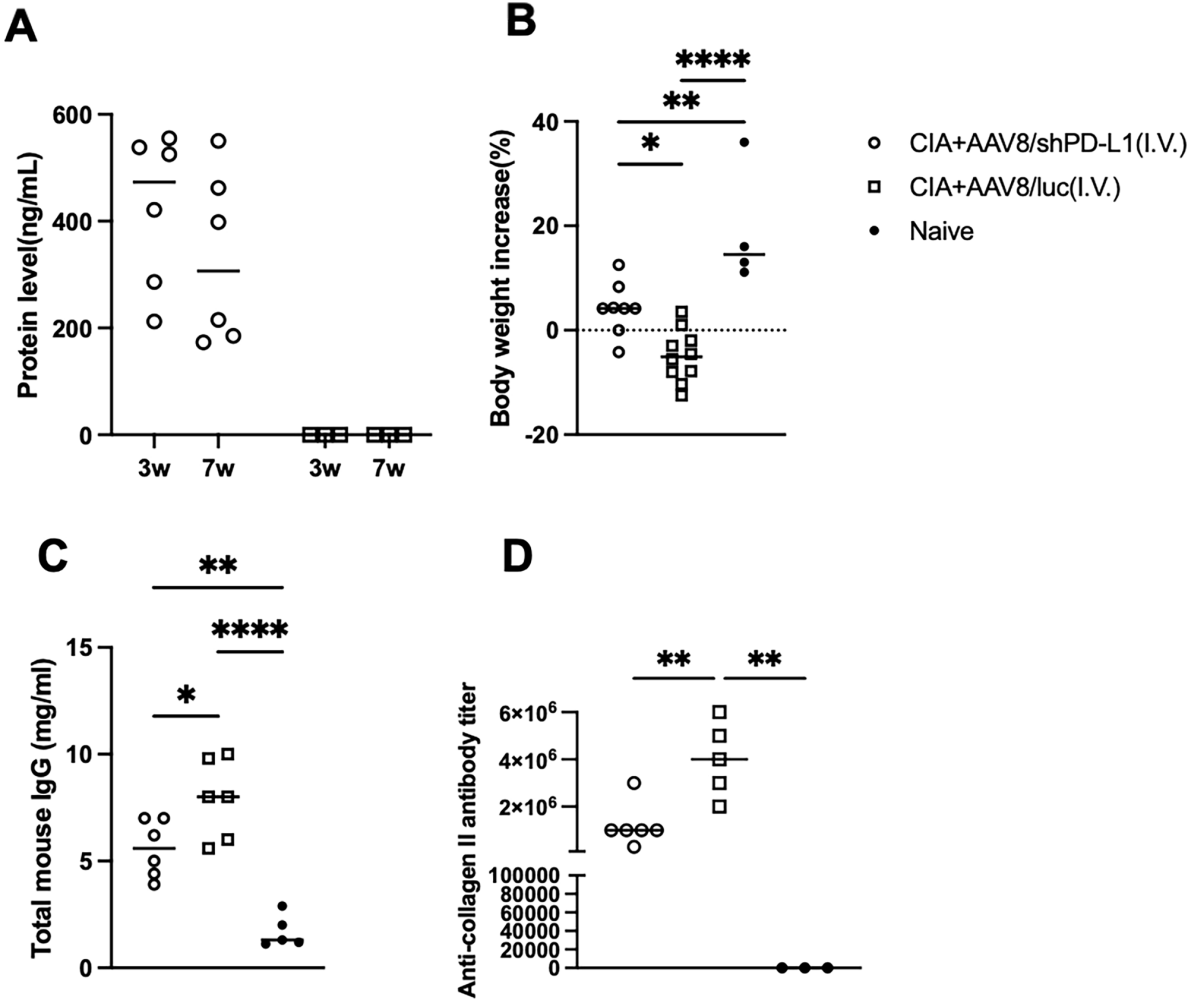
Overall symptoms and corresponding antibody levels in CIA mice intravenously injected with AAV8/shPD-L1. **(A)** Protein level of shPD-L1 in the serum of mice at 3- and 7-weeks post-injection (n=6). Data were analyzed using two-tailed unpaired Student’s t test. **(B)** Body weight changes from 0w to 7w between CIA mice intravenously injected with AAV8/shPD-L1(n=10), AAV8/luc(n=10), and naïve mice(n=4). **(C)** Total mouse IgG level between CIA mice intravenously injected with AAV8/shPD-L1(n=6), AAV8/luc(n=6), and naïve mice(n=5). **(D)** Anti-collagen II antibody level between CIA mice intravenously injected with AAV8/shPD-L1(n=6), AAV8/luc(n=5), and naïve mice(n=3). Data from panels **(A–D)** were analyzed using one-way ANOVA followed by Bonferroni multiple comparison test for group comparisons. *p < 0.05, **p < 0.01, ****p < 0.001.

The protein levels of shPD-L1 in the blood were examined at week 3 and week 7 post injection and were 422.8 ± 144.5 ng/mL at week 3 and 330 ± 160.8 ng/mL at week 7. No significant difference was observed in the average protein levels between week 3 and week 7 and no PD-L1 was detected in the AAV8/luc mice (**Figure 4A**) (20). Interestingly, the body weights of the mice in each group exhibited a distinct trend. The CIA control mice without shPD-L1 showed a significant decrease in body weight (%) (-4.2 ± 5.0), while the treated group showed a slight increase in body weight (4.9 ± 5.0), and the naïve mice showed a significant increase in body weight (19.0 ± 11.5) (**Figure 4B**). These data manifested that intravenous AAV administration affected clinical symptoms, including body weight, paw swelling, and joint inflammation.

### Decreased levels of total IgG and collagen-specific IgG in CIA mice intravenously injected with AAV8/shPD-L1

In terms of the total mouse IgG level, control CIA mice treated with AAV8/luc showed significantly higher IgG levels, approximately 1.5-fold higher than CIA mice intravenously treated with AAV8/shPD-L1, and 4-fold higher than naïve mice (7.9 ± 1.88mg/ml vs 5.6 ± 1.38mg/ml vs 1.7 ± 0.8mg/ml) (**Figure 4C**). Moreover, we also analyzed the levels of anti-collagen antibodies. In naïve mice, no antibodies were detected. By week 7, we observed a 2-3-fold lower anti-collagen antibody level in CIA mice with AAV8/shPD-L1 treatment compared to those without treatment (1.2x10^6^ ± 9.1x10^5^ vs 3.8 x10^6^ ± 1.6 x10^6^) (p <0.05) (**Figure 4D**). This evidence indicated that intravenous AAV administration altered the systemic antibody profiles.

### Reduced cytokine levels in serum from CIA mice intravenously injected with AAV8/shPD-L1

**Figure 5:**
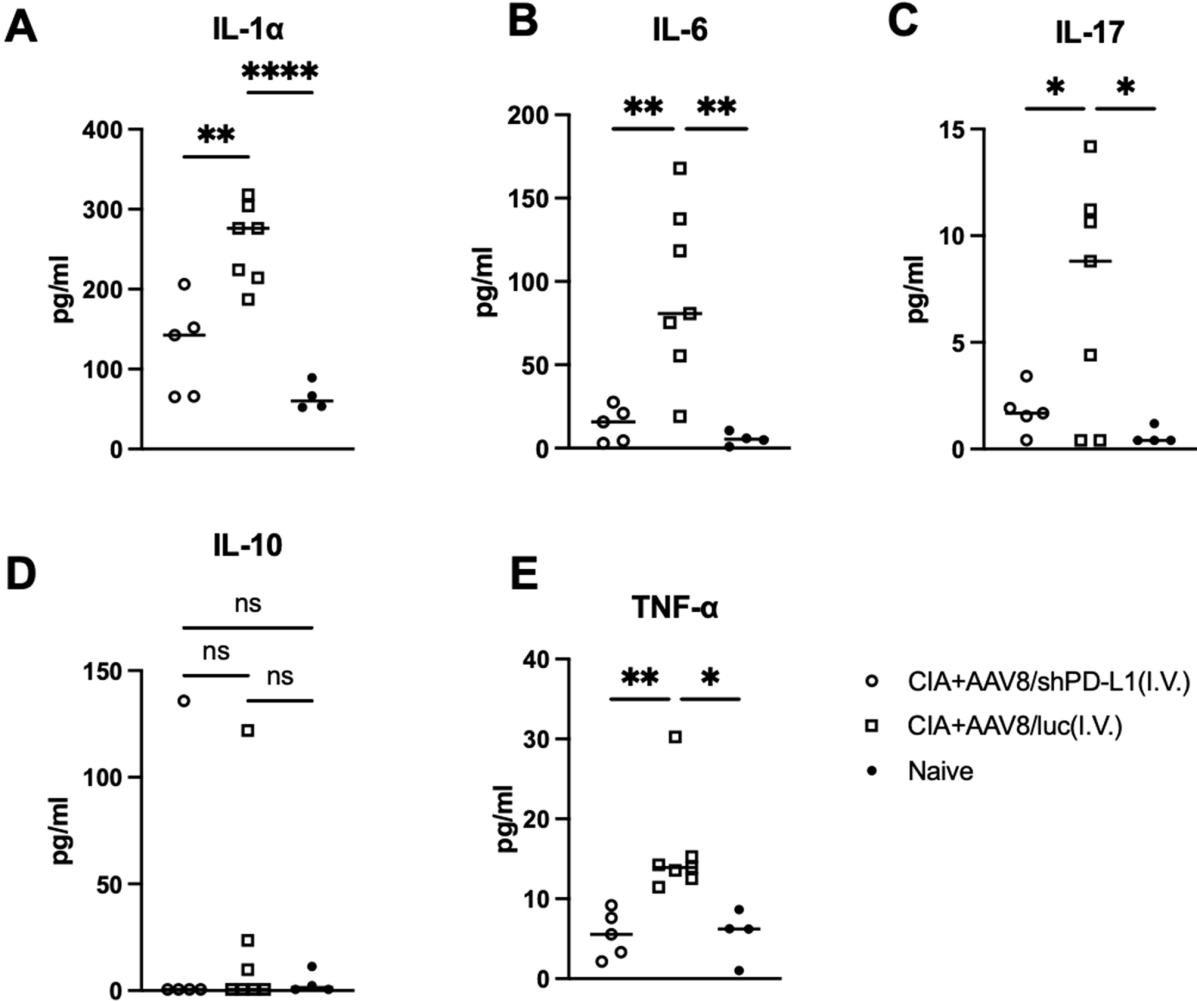
Cytokine levels in serum from mice intravenously injected with AAV8/shPD-L1. 7w after CIA induction, serum from mice injected with AAV8/shPD-L1(n=5), AAV8/luc (n=7), and naïve mice (n=4) were collected, IL-1α **(A)**, IL-6 **(B)**, IL-17 **(C)**, IL-10 **(D)**, and TNF-α **(E)** were detected using cytokine multiplex kit. Data were analyzed using one-way ANOVA followed by Bonferroni multiple comparison test for group comparisons. *p < 0.05, **p < 0.01, **** p < 0.001.

Seven weeks after CIA induction, serum from mice injected with AAV8/shPD-L1, AAV8/luc, and naïve mice were collected and the levels of IL-1α, IL-6, IL-17, IL-10, and TNF-α were measured. Compared to those treated with shPD-L1, the levels of IL-1α in CIA AAV8/luc treated mice were found to be 2.5-fold higher (126.3 ± 60.5 vs 257 ± 49.1), IL-6 8-fold higher (14.3 ± 10.6 vs 93.5± 51.0), IL-17 4-fold higher (1.8 ± 1.1vs 7.1 ± 5.5), and TNF-α 3-fold higher (5.6 ± 2.9 vs 15.9 ± 6.5). However, there was no significant difference in the level of IL-10 between these groups (**Figure 5**).

### Intravenous injection with AAV8/shPD-L1 altered immune cell profile in the spleen of CIA mice

**Figure 6:**
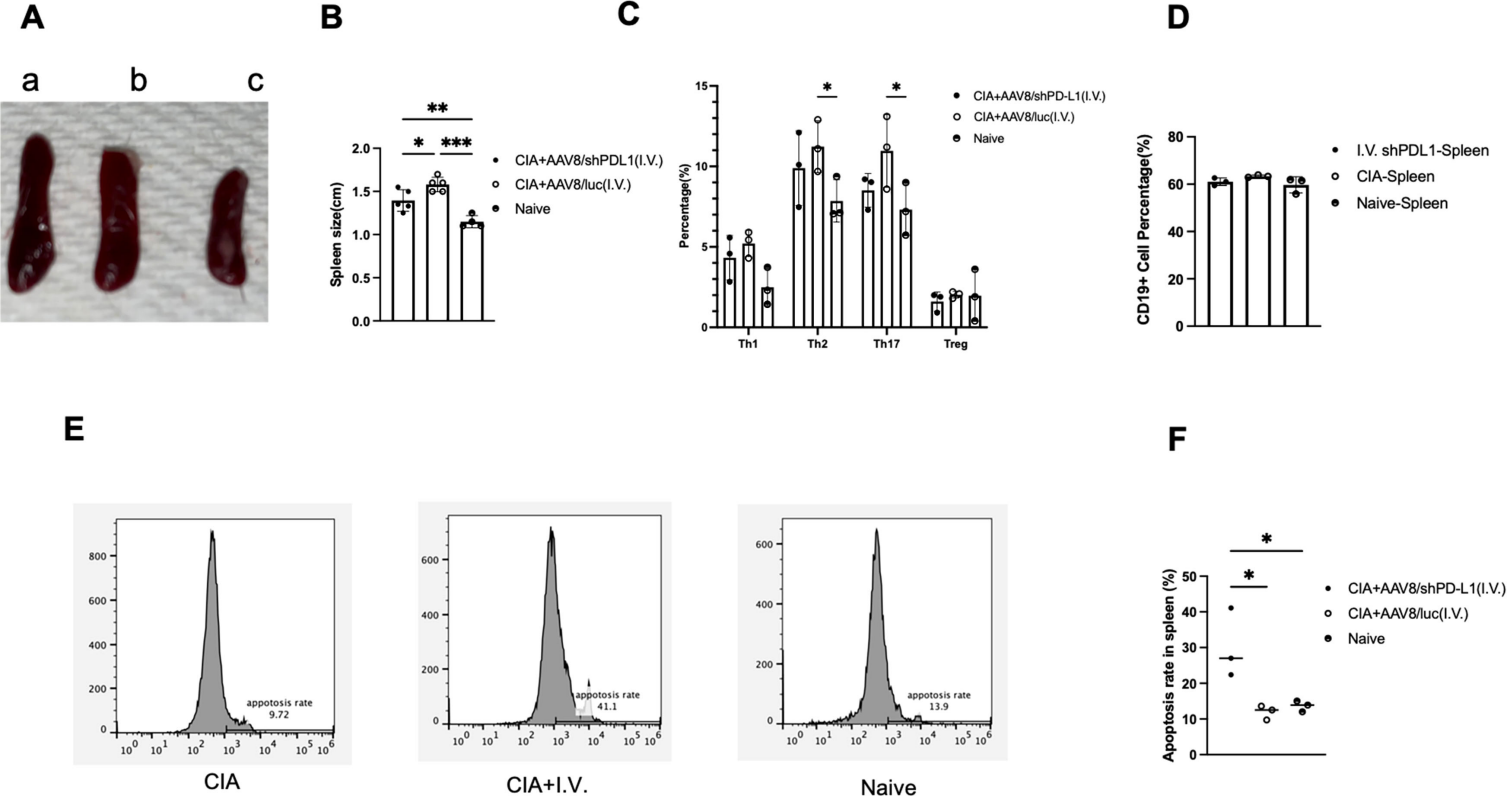
Intravenous injections with AAV8/shPD-L1 altered immune cell profile in spleen. **(A)** Representative image of size comparison of spleens. a, CIA+I.V. AAV8/luc, b, CIA+I.V. AAV8/shPD-L1, c, naïve. **(B)** Quantification of spleen size(n=5). **(C)** the percentages of T cell subsets between CIA mice intravenously injected with AAV8/shPD-L1, AAV8/luc, and naïve mice(n=3). **(D)** CD19+ cells percentage between CIA mice intravenously injected with AAV8/shPD-L1, AAV8/luc, and naïve mice(n=3). **(E)** Apoptosis rate of T cells between CIA mice intravenously injected with AAV8/shPD-L1, AAV8/luc, and naïve mice. **(F)** Summary of apoptosis in spleen (n=3). Apoptosis rates per group were determined by gating for the shift in the Annexin V-positive population in the flow cytometry histograms. Data from panels **(B, C, D, F)** were analyzed using one-way ANOVA followed by Bonferroni multiple comparison test for group comparisons. *p < 0.05, **p < 0.01, ***p < 0.005.

Since shPD-L1 is a potential immunosuppressant, we further analyzed the systemic immune response following shPD-L1 injection. We specifically focused on the spleen as it is one of the largest and most important lymphoid tissues in the body. We observed that spleen sizes were the largest in control treated CIA mice, followed by CIA mice treated with shPD-L1, and then naïve mice (**Figures 6A, B**). Additionally, levels of Th2 (%) (11.2 ± 1.6 vs 7.9 ± 1.3) and Th17 cells (%) (11.0 ± 2.3 vs 7.3 ± 1.6) were found to be higher in CIA mice (**Figure 6C**) compared to naïve mice (p <0.05). There was no observed difference in B cell percentages, with a positive rate of around 60% in each group (**Figure 6D**). We further assessed the apoptosis rate of T cells in the spleen among those groups and found that the T cell apoptosis rate (%) was also 2-fold higher in mice treated with shPD-L1(30.2± 9.7) compared to CIA mice without treatment (11.9 ± 2.0) and naïve mice (13.6 ± 1.5) (**Figures 6E, F**). This finding further demonstrated that shPD-L1 could induce the immune cell apoptosis thus decrease the activated immune cells systemically.

### Intramuscular injection with AAV6/shPD-L1 improved overall symptoms and corresponding biomarkers in CIA mice

One concern with intravenous delivery of shPD-L1 is its potential overexpression in the liver. The high expression of shPD-L1 in the liver might cause liver toxicity and immune suppression, which further facilitates infections and even tumorigenesis (21).

**Figure 7:**
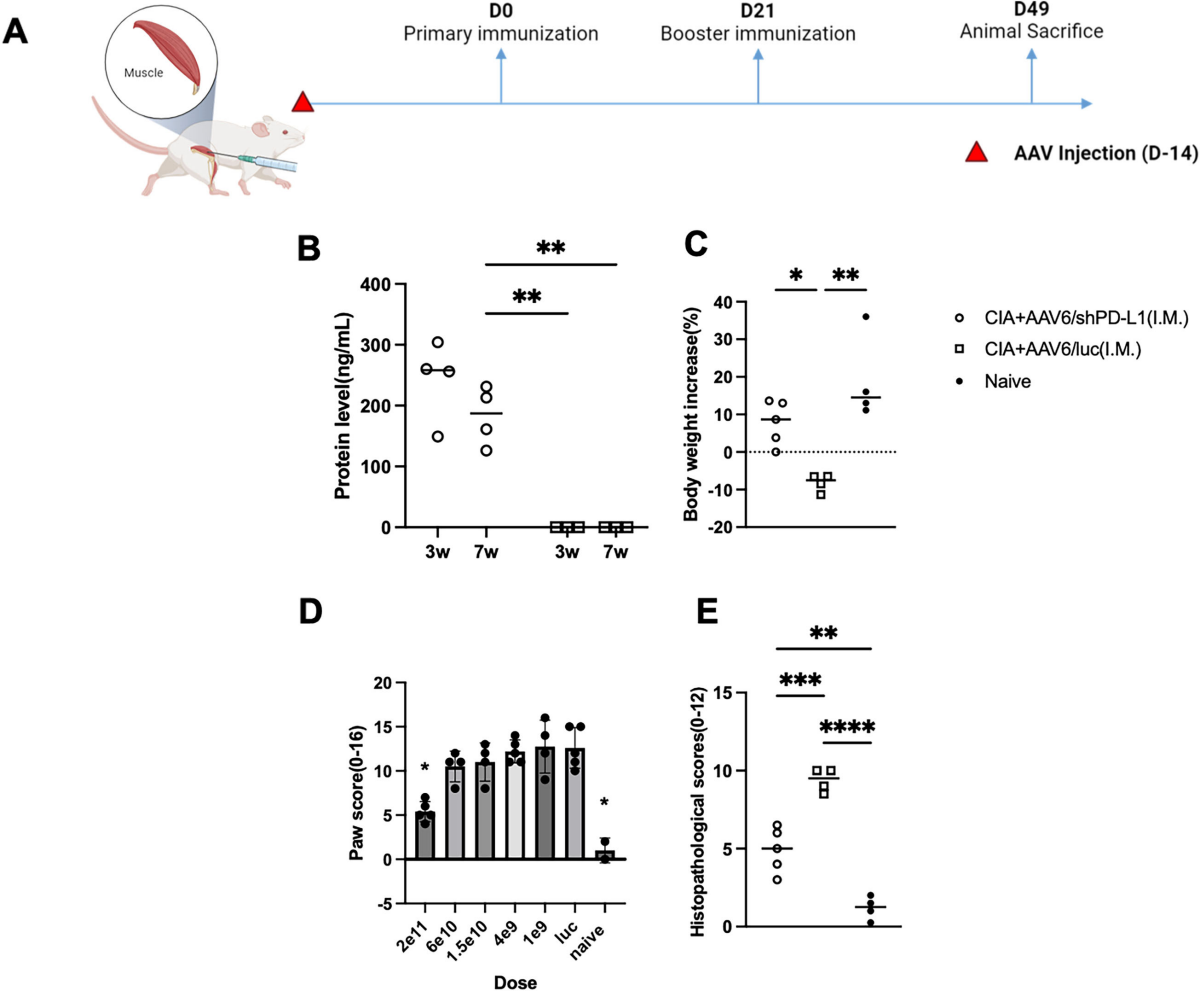
Effects of intramuscularly injected AAV6/shPD-L1 in CIA mice. **(A)** Diagram of mouse injections. Mice were injected intramuscularly 2 weeks prior to primary immunization on day 0. Booster injections were performed on day 21, and mice were sacrificed on day 49. **(B)** Protein level of shPD-L1 in mice serum at 3w and 7w(n=4). Mice were injected with 2e11vg of AAV6/shPD-L1 or AAV6/luc, and serum was collected at 3w and 7w. Data were analyzed using two-tailed unpaired Student’s t test. **(C)** Body weight change between CIA mice intramuscularly injected with AAV6/shPD-L1(n=5), AAV6/luc(n=4), and naïve mice(n=4). **(D)** Paw score of mice treated with different doses (n=5) of AAV6/shPD-L1, AAV6/luc, and naïve mice. **(E)** Histological scores of CIA mice intramuscularly injected with AAV6/shPD-L1(n=5), AAV6/luc(n=4), and naïve mice(n=4). Data from panels **(B-D)** were analyzed using one-way ANOVA followed by Bonferroni multiple comparison test for group comparisons. *p < 0.05, **p < 0.01, ***p < 0.005, ****p < 0.001.

Based on our *in vivo* imaging, we used both AAV6/luc and AAV8/luc for systemic delivery. We found that the luciferase signal from intravenously delivered AAV8 primarily localized to the liver, whereas intramuscularly injected AAV6 remained mainly at the injection site (**Supplementary Data**). Therefore, we explored systemic treatment via intramuscular injection (IM) using AAV6, as it is the most effective serotype for muscle transduction and less liver transduction (22) (**Figure 7A**). We administrated the AAV6/shPD-L1 vector to the mice intramuscularly in both legs at a dose of 2x10^11^ vg/mice in a total volume of 100 µL. Throughout our observation period, we detected that the protein levels in injected mice sera remained over 200 ng/mL (**Figure 7B**). The body weight changes (%) in AAV6/shPD-L1 treated CIA mice, AAV6/luc treated CIA mice, and naïve mice were 7.8 ± 5.9, -8.2 ± 2.3, and 19.0 ± 11.5, respectively (**Figure 7C**). There was around a 50% improvement in paw swelling (**Figure 7D**) and joint histology (**Figure 7E**) as well as the decline of antibodies and inflammatory cytokine levels (**Supplementary Data**). This delivery method also conveys an advantage of negligible genome expression in the liver (data not shown), compared to intravenous injection which results in strong gene expression in the liver.

We further compared the dose response of intramuscular treatment with six doses of 2x10^11^, 6x10^10^, 1.5x10^10^, 4x10^9^, 1x10^9^, and 0 vg, with a total volume of 100 µL in each mouse, five mice were included in each dose group. Seven weeks after CIA induction, the paw swelling in each mouse was scored independently by two observers and averaged. Our finding indicates a dose-dependent effect. Specifically, the highest dose, 2e11vg, partly mitigated the paw swelling (5.4 ± 1.1 vs 10 ± 2.0) and joint inflammation (4.9 ± 1.4 vs 9.3 ± 0.8) compared to AAV6/luc treated mice (p <0.05), whereas the lower doses failed to produce any improvement with no significant difference between those groups and control treated mice (**Figure 7D**). Based on the dose-response analysis, intramuscular injection appears to be a safer option; however, its efficiency may still require further optimization.

## Discussion

RA is a complex systemic autoimmune disorder that primarily targets the joints. Various biologic treatments have been explored and face challenges in maintaining drug pharmacokinetics and stabilizing protein or chemical concentrations within the joint, including intra-articular delivery (23). Compared to those treatments, gene therapy with AAV vectors offers sustainable potency while maintaining a favorable safety profile (23). Previously, we explored the intra-articular injection of AAV encoding wild type PD-L1, which provided partial relief from arthritis and locally blocked immune cell infiltration (13). In the present study, we engineered a soluble PD-L1 variant (shPD-L1) with strong secretion capability that exhibited greater efficacy than wild type PD-L1 when administered at the same dose via intra-articular injection. We also demonstrated the therapeutic effect of systemic expression of shPD-L1 by delivering AAV vectors encoding shPD-L1 via intravenous and intramuscular routes. Specifically, intravenous injection nearly restored inflammation to naïve phenotype levels, and intramuscular injection also resulted in therapeutic benefits in the overall findings of inflammatory arthritis.

Intra-articular injection has been a common route of gene delivery for local gene therapy in arthritis due to precise effects with fewer adverse events. Several serotypes, including AAV5 and AAV6 (24), among others, have been used as viral vectors to deliver transgenes intraarticularly, as they demonstrated limited liver transduction and ideal transduction in the joint (25, 26) when compared to other AAV serotypes. In our study, similar to the results from previous reports using AAV5 (27), shPD-L1 expression via intra-articular injection was exclusively detected in AAV6 vector-transduced joints. This study demonstrated that shPD-L1 effectively blocked immune cell infiltration and decreased inflammation in the arthritic knee joints following local injection. It is worth noting that the collagen antibody titers in serum were not altered, and shPD-L1 protein expression in serum was not detected (data not shown). These results further support the conclusion that the effects of shPD-L1 from AAV vector intra-articular injection were confined within the local joint area and indicate a good safety profile for intra-articular injection of AAV vectors for arthritis treatment.

However, intra-articular injection also faces some challenges. One limitation is their limited ability in treating multiple joints simultaneously with one single intra-articular injection. Though some patients exhibit arthritis in only one or a few joints or at the least a flare in only one joint, polyarthritis is still the most common presentation in various inflammatory joint-related disorders such as RA. Additionally, RA is a systemic disease characterized by circulating auto-antibodies and activated immune cells in the systemic circulation. Other tissues besides articular joints such as the lungs and heart are also involved in 10-20% of RA patients. Intra-articular administration of AAV vectors can effectively block lymphocytes in the joints, however, local PD-L1 expression is not able to prevent circulating antibodies from migrating into the synovial fluid to induce joint damage, then diminishing the treatment’s overall efficacy.

To overcome the shortcomings of intra-articular injection, we further explored the therapeutic effect of systemic expression of shPD-L1 from AAV gene therapy in CIA mice. The common routes for systemic delivery are intravenous injection (IV) and intramuscular injection (IM). Among them for AAV delivery, intravenous injection has been shown to provide a rapid onset of expression and a broad distribution of targeted genes based on AAV serotypes, as the AAV vector can circulate in the blood, access the central compartment, and effectively bypass the digestive system (28). In our study, intravenous injection of AAV8/shPD-L1 vectors induced a high efficacy with improvements in joint histology and paw scores in CIA mice, almost reaching that in the naïve mouse, along with a significant drop in pro-inflammatory cytokine and auto-antibody levels in sera. The spleen also showed a significant increase in T cell apoptosis rate. These results indicate that intravenous injection altered the systemic immune response, impacting the overall systemic inflammation.

Based on the results from clinical trials, liver targeting by IV administered AAV raises several safety concerns. When administered systemically, AAV vectors tend to accumulate in the liver due to its high blood flow (29). Liver toxicity has been reported in 30-50% of patients, with some cases resulting in fatal outcomes (30). Therefore, we explored an alternative approach for systemic transgene expression via intramuscular administration of AAV vectors. Compared to IV, the IM route was a more feasible and less invasive route (31–33). This method effectively transduces muscle cells and when secretory proteins are used, as in this study, the secreted proteins can be effectively distributed into the bloodstream. The IM route has been successfully employed in treating various diseases in preclinical studies, such as hemophilia and metabolic diseases (34). However, a disadvantage of IM was lower transgene expression compared to IV. In our study, shPD-L1 levels in the blood from IM were approximately 50% lower than that from IV, leading to reduced efficacy. This indicates the necessity to explore effective strategies to enhance shPD-L1, for example, optimization of the shPD-L1 codon, utilization of strong promoters, or engineering AAV capsid for high muscle tropism. Another strategy could be to inject in multiple sites or muscles, since one muscle injection of AAV vectors at a high dose may lead to saturation of protein expression. Additionally, combining shPD-L1 with another type of inhibitory protein, such as CTLA4, could potentially enhance the treatment’s efficacy.

In this study, systemic expression of shPD-L1 impacts systemic immune responses. The antibody levels in sera, including both total IgG and anti-collagen II, were 2-3-fold lower in treated mice than that in control CIA mice without treatment. Interestingly, the arthritic symptoms in mice treated with IV AAV8/shPD-L1 were similar to the phenotype in naïve mice, but the antibody levels including total IgG and collagen-specific IgG in treated mice remained significantly higher than those in the naïve mice. This implies that partially blocking the inflammation and immune response may be sufficient to manage the disease and improve the symptoms. This finding is consistent with reports that some patients in the early phase show biomarker upregulation without displaying symptoms. Among the cytokines we observed, the most significant difference was observed in TNF-α and IL-6 levels although all of the proinflammatory cytokines were significantly decreased in mice treated with IM and IV shPD-L1 compared to control treated mice. These two cytokines have already been largely reported and established as important single pharmaceutical targets (35). Due to limited resources, we did not investigate other immune cells besides B cells and T cells, such as macrophages, neutrophils, and monocytes. However, it is possible that shPD-L1 treatment may have also affected these other immune cell populations. The reduction in both antibodies and cytokines was more pronounced in the CIA mice receiving IV when compared to IM (**Supplementary Data**), closely related to phenotypic correction of arthritis. One direct factor contributing to this difference is the level of shPD-L1 expression. Another potential reason may be due to locally high PD-L1 expression from liver targeting of AAV vectors and high volume of blood that will provide more opportunity for interaction of shPD-L1 and immune cells and then execute a greater potential to regulate an immune response. While an appropriate amount of PD-L1 can effectively inhibit excessive inflammation, excessive PD-L1 could contribute to immune evasion, potentially leading to tumorigenesis (36). In our study, we did not observe any significant abnormalities or tumor growth in the liver, spleen, heart, lung, kidney, or intestine. However, it is crucial to monitor the immune status and minimize the risk of tumorigenesis. The strategies include determining the optimal PD-L1 dose to balance immune stimulation and suppression, localizing PD-L1 expression to the desired tissue and reducing PD-L1 levels in the liver and other tumor-prone organs using tissue-specific promoters or modified AAV capsids. Additionally, using an inducible promoter for PD-L1 expression could ensure that PD-L1 is only expressed during promoter activation.

In conclusion, this paper studied the efficacy of multiple delivery routes for AAV-mediated shPD-L1 delivery in the treatment of RA using the mouse CIA model. We found that shPD-L1, delivered via intra-articular injection of AAV vectors, demonstrated a more potent therapeutic effect compared to wild type PD-L1. Intravenous injection for systemic expression of PD-L1 was effective in blocking the progression of arthritis, while intramuscular injection emerged as a promising and safe alternative. Future work will aim to optimize transgenes and AAV capsids to enhance transgene expression when muscle tissues are targeted for systemic expression of shPD-L1.

## Materials and methods

### Cell culture and AAV vector production

HEK-293 cells were grown in Dulbecco’s Modified Eagle Medium (Thermo Fisher, Waltham, Massachusetts, US) supplemented with 10% fetal calf serum, 100 μg/mL of penicillin G, and 100 μg/mL of streptomycin at 37°C. The cells were regularly passaged at a 1:5 ratio three times per week after reaching approximately 90% confluence.

To produce AAV vectors, HEK-293 cells were triple transfected. Cells and supernatants were purified using a cesium chloride (CsCl) ultracentrifugation gradient. AAV titers were measured using quantitative real-time polymerase chain reaction (qPCR) at a 10 μL volume in 96-well plates, detected using the Fast SYBR Green Master Mix (Applied Biosystems, Foster City, California, USA). AAV vector genome integrity was confirmed via alkaline gel electrophoresis. SYPRO Ruby protein gel stain (Thermo Fisher, Waltham, Massachusetts, US) was used to verify the capsids contained all three VP1, VP2 and VP3 proteins. Details were previously described in Li et al., 2024 (24).

### Construction of AAV cassette for soluble PD-L1 protein expression

The cDNA of PD-L1 variants (shPD-L1, hPD-L1, and secPD-L1) was synthesized and cloned into the pTR-CBh-PD-L1 backbone using the restriction enzymes HindIII and NotI, as well as Golden Gate assembly. The expression was driven by the CBh promoter, which has been characterized by robust, long-term, and ubiquitous transgene expression (37). The 6* His-tag (CACCATCACCATCACCAT) was fused to PD-L1 variants directly upstream of the stop codon. The three variant sequences were confirmed through whole plasmid sequencing.

### Western blot for PD-L1 expression

The pTR-CBh-PD-L1 plasmid was transfected into HEK-293 cells cultured in a 6-well plate. After 48 hours, both supernatants and cell lysates were harvested. GAPDH and beta-actin were used as loading control. Details were previously described in Li et al., 2023 (13).

### T cell assays for PD-L1 function

shPD-L1 protein was purified from shPD-L1-transfected HEK-293 cells. The supernatant and cell lysates were collected and shPD-L1 protein was purified using a HisTrap column (Cytiva, MA, USA); Pan T cells from mouse spleen were stained with CellTrace Violet dye as indicated by the CellTrace™ Violet Cell Proliferation Kit (Thermo Fisher, Waltham, Massachusetts, US), then incubated with 2x10^5^ anti-CD3/CD28 beads (Thermo Fisher, Waltham, Massachusetts, US) and 10U/ml IL-2 (R&D, MN, USA) with or without 5 μg/ml of purified PD-L1. Recombinant PD-L1 and PBS were used as positive and negative controls, respectively. T cells cultured with no anti-CD3/CD28 beads were also designed. After 72h, the percentage of proliferating T cells from each group were determined by Attune Flow Cytometer (Thermo Fisher, Waltham, Massachusetts, US) with an emission of 405/445nm. Details were previously described in Li et al., 2023 (13).

### Collagen-induced arthritis mouse model

All animal care and housing requirements were followed under the guidance of the National Institutes of Health Committee on the Care and Use of Laboratory Animals of the Institute of Laboratory Animal Resources, and all animal protocols were reviewed and approved by the Institutional Animal Care and Use Committee at the University of North Carolina at Chapel Hill. Male DBA/1J mice at the age of 7-8 weeks were used to mimic the acute inflammatory conditions of RA (38).

Two immunization doses of bovine type II collagen (Chondrex, Woodinville, WA, USA) were injected at the root of the mouse tail at day 0 and day 21 respectively. Details were previously described in Li et al., 2023 (13).

### Animal study design

For local treatment, AAV was injected on the same day as the primary immunization with type II collagen. One group of mice received intra-articular administration of self-complementary (sc) AAV6/PD-L1 driven by the CBh promoter at different doses in a total volume of 5 μl in the left knee joint. AAV6/shPD-L1 was applied in the contralateral right knee joint as a control. The positive control group consisted of AAV6/luc. The negative control group consisted of naïve mice. For systemic treatment, two injection routes were applied two weeks before primary immunization: intravenously via the retro-orbital (RO) venous sinus using a total volume of 100 μl, and intramuscularly in the hindlimb muscle in a total volume of 100 μl.

### Paw swelling measurement

To evaluate paw swelling, mouse paws and toes were evaluated and scored by 2 independent, blinded observers. Each paw was assessed using a 4-point scale and the scores from all four paws were added together to get the total score for each mouse. The scoring scale is as follows: 0 indicates normal; 1 indicates redness in one or two toes; 2 indicates redness or swelling in more than two toes; 3 indicates swelling of the entire paw; and 4 indicates severe swelling or ankylosis. Thus, the total scoring scale is from 0 to 16 (39).

### Tissue histopathology

After 7 weeks post-primary CIA immunization, the mice were sacrificed, and knee joints were collected by dissecting the femur and tibia 5 mm from the knee joint. Harvested knees were processed for H&E. Details were previously described in Li et al., 2023 (13). Histopathology scores were on a 12-point scale and were based on the following four conditions: synovial hyperplasia (0–3), infiltration of leukocytes into the synovial membrane/joint space (0–3), pannus formation (0–3), and the necrosis/erosion of cartilage (0–3) (40).

### Cytokine assay

Following mouse euthanization on week 7 post-primary CIA immunization, sera were analyzed by first collecting blood from the retro-orbital plexus using non-heparinized micro-hematocrit capillary tubes (DWK Life Sciences, Millville, NJ, US) from each mouse. The blood was then set for 30 min at room temperature. The blood was centrifuged at 3,000 rpm for 10 min and the supernatants were collected. Total protein concentration was measured through BCA assay. Multiple cytokines in the knee joint homogenization, including IL-1, IL-6, IL-17A, TNF-α, and IL-10, were measured using a Luminex MAGPIX system (Luminex Corporation, Austin, TX, USA). Cytokine levels were expressed in picograms per milliliter (pg/ml), and levels below the detection limit were defined as 0 pg/ml for each cytokine. The cytokine levels per mg of protein were calculated.

### Flow cytometry

The mouse spleens were processed into a single-cell suspension, strained through a 70 µm cell strainer, followed by red blood cell lysis using ACK buffer and 2 washes with PBS. For the apoptosis assay, CD3-APC (BD Biosciences, Franklin Lakes, NJ, US) and Annexin V-FITC (BD Biosciences, Franklin Lakes, NJ, US) were used to stain the spleen cells, followed by flow cytometry analysis. To stain the T cell subsets, splenocytes were stimulated at approximately 1e6 cells/mL with 2 µL of Leukocyte Activation Cocktail and 2 µL of GolgiPlug (BD Biosciences, Franklin Lakes, NJ, US) per mL of culture, followed by a 4-hour incubation at 37°C. Following stimulation, surface staining was performed using anti-mouse CD4-FITC antibody (BD Biosciences, Franklin Lakes, NJ, US), after which cells were fixed and permeabilized using the Fix/Perm kit (eBioscience, San Diego, CA, US) and stained for the following intracellular markers: IFNγ-eFluor660, IL-4-PE, IL-17-eFluor, CD25-eFluor, and FOXP3-PE. The stained cells were then analyzed using an Attune flow cytometer (Thermo Fisher, Waltham, Massachusetts, US) and the data was processed and interpreted using FlowJo software.

### Detection of antibodies

To detect anti-collagen II antibodies in sera, 50 ng/μl type II collagen was mixed with 100 μl 1x coating buffer (BioLegend, San Diego, CA, US) and coated on a Corning Costar Brand 96-Well EIA/RIA Plate (Thermo Fisher, Waltham, Massachusetts, US) overnight. Details were previously described in Li et al., 2023 (13). To detect total mouse IgG, a commercial kit was used (Abcam, Cambridge, UK). OD value was measured at 450nm.

### Statistical analysis

Statistical analysis was conducted using GraphPad Prism 9 software. Results are presented as mean ± SD, with descriptive statistics depicted using box and whisker plots. Group differences were assessed through one-way ANOVA or Student’s t test, with Bonferroni and Sidak tests employed for multiple comparisons between groups. A significance level of 0.05 was utilized. Based on power analysis of our preliminary data using nQuery software, the power of mouse sample size exceeded 80% at a significance level of 0.05.

## Data Availability

The original contributions presented in the study are included in the article/**Supplementary Material**. Further inquiries can be directed to the corresponding author.
