# Supplementary material for: Secreted PD-L1 alleviates inflammatory arthritis in mice through local and systemic AAV gene therapy
Source: Front Immunol. 2025 Feb 3;16:1527858. doi: 10.3389/fimmu.2025.1527858 (PMC11830590; doi:10.3389/fimmu.2025.1527858)
Supplement: Supplementary file 1 [file DataSheet1.pdf]

The International Society for the Advancement of Cytometry (ISAC) has highlighted the importance of including comprehensive methodological information to ensure data reproducibility and reliability. In line with this, Frontiers in Immunology now requires authors to submit a checklist for manuscripts that involve flow or mass cytometry. This checklist helps standardize the reporting process, improving the quality and transparency of published data. By doing so, we support scientific progress, making it easier for other researchers to replicate and validate experiments.

This form should be submitted with any manuscripts using flow or mass cytometry.

Sample/specimen/material description

☐ Total blood

☐ PBMCs

☑ Organ digests

Other____

Did the samples suffer any treatment before or after incubation with the antibodies?

☑ Drug AAV

☐ Cell permeabilization ______________________

☐ Dye _____________________________________

☐ Propidium iodine

☐ Not applicable

Other _____________________________________

Instrument and antibodies

Name of the Cytometer attune

Antibodies and targets	Fluorochrome/ Metal	Catalog number/Company
e.g. anti-CD4	FITC	Cat. XXX/ XXX ltd.
CD4	FITC	BD Biosciences

CD3	APC	BD Biosciences
Annexin V	FITC	BD Biosciences
IFNγ	eFluor660	BD Biosciences
IL-4	PE	BD Biosciences
IL-17	eFluor	BD Biosciences
CD25	eFluor	BD Biosciences
FOXP3	PE	BD Biosciences

Data analyses

1. Name of the software Flowjo
2. Reference gating strategy in the manuscript or supplementary material

Gating strategy in (eg Figure X) Fig. 6
