# Supplementary material for: Secreted PD-L1 alleviates inflammatory arthritis in mice through local and systemic AAV gene therapy
Source: Front Immunol. 2025 Feb 3;16:1527858. doi: 10.3389/fimmu.2025.1527858 (PMC11830590; doi:10.3389/fimmu.2025.1527858)
Supplement: Supplementary file 2 [file DataSheet2.docx]

**
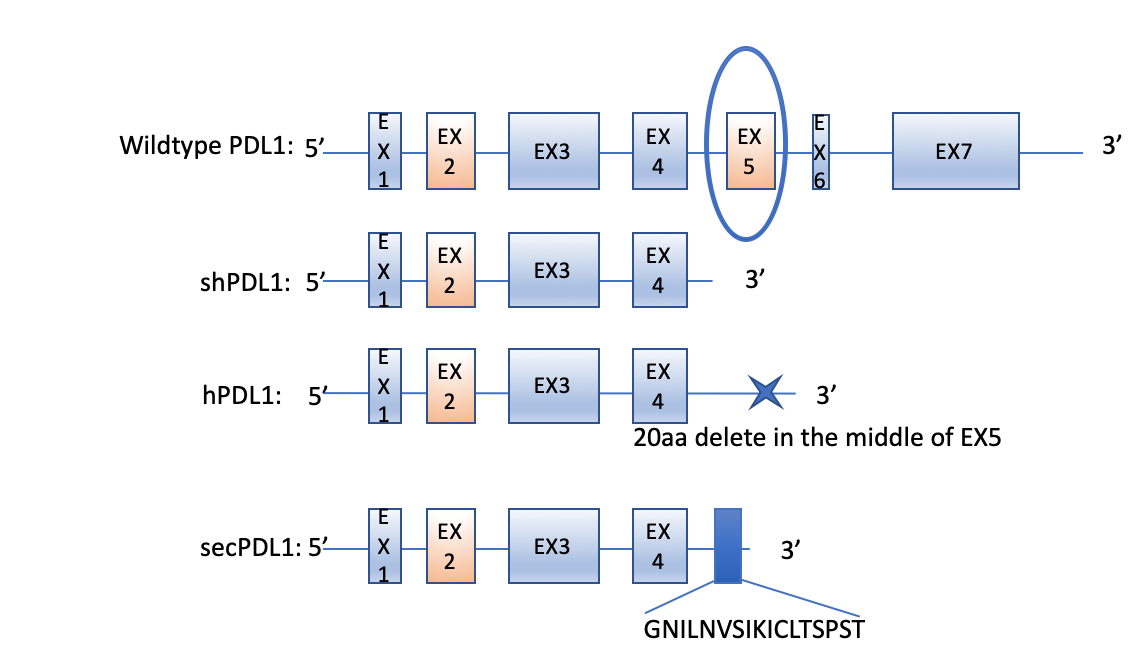
**

**Fig. S1 The schematic sequence structure of wild type PD-L1 and PD-L1 variants.**

**Fig. S2 Paw score of mice intra-articularlly treated with AAV6/shPD-L1, AAV6/PD-L1, AAV6/luc, and naïve mice.** The paw swelling score(n=5) was assessed independently by two observers, with each of the four paws receiving a score ranging from 0 to 4. The total score for each mouse was calculated by summing the individual paw scores. Data were analyzed using one-way ANOVA followed by Bonferroni multiple comparison test for group comparisons. *p < 0.05.

**Fig. S3, The percentages of T cell subsets in spleen between CIA mice intra-articularlarly injected with AAV6/shPDL1, AAV6/luc, and naïve mice.** Data were analyzed using one-way ANOVA followed by Bonferroni multiple comparison test for group comparisons.

**Fig. S4 Anti-collagen II antibody titer from mice intra-articularly injected with AAV.** 7w after CIA induction, mice serum was collected and diluted from 1:10 to 1:10^7^ with three times dilution. The antibody titer was determined by the OD value that was 3 times higher than that in naïve mice. Data were analyzed using one-way ANOVA followed by Bonferroni multiple comparison test for group comparisons.


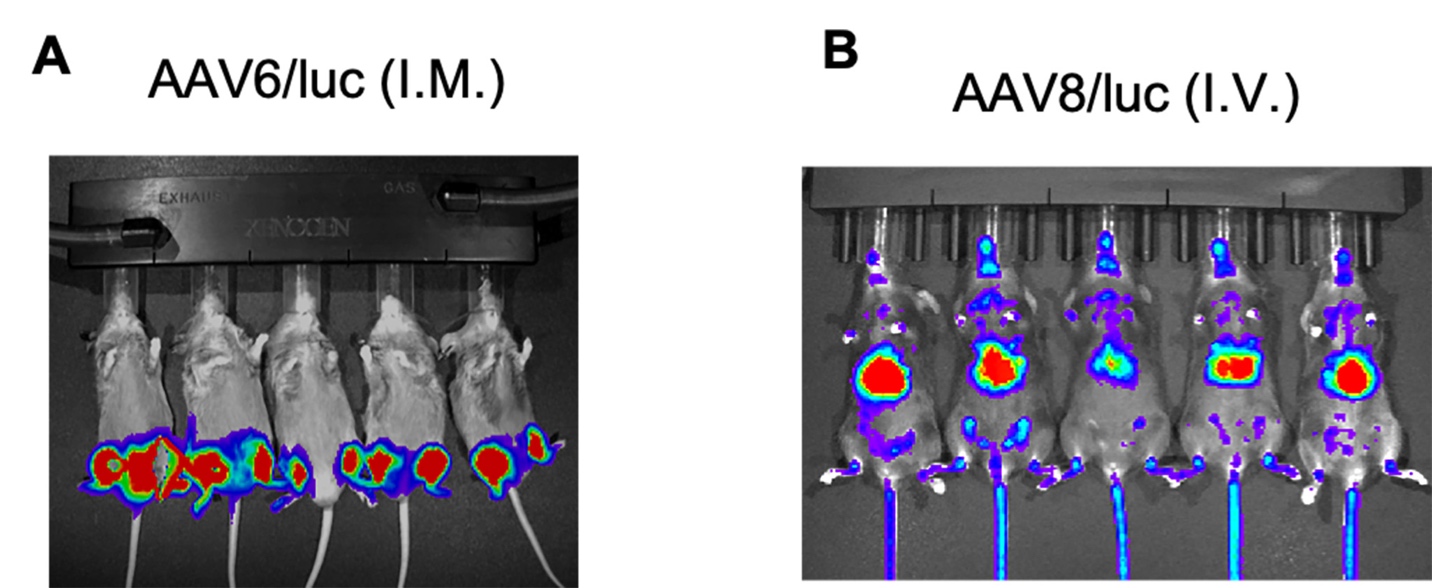


**Fig. S5, In vivo imaging of luciferase signal.** A, AAV6/luc vector was administered to the mice intramuscularly in both legs at a dose of 2x10^11^ vg/mice in a total volume of 100 µL. B, AAV8/luc vector was administered to the mice retro-orbitally at a dose of 2x10^11^ vg/mice in a total volume of 100 µL.

**Fig. S6 Cytokine levels in CIA mice treated with intramuscular AAV6/shPD-L1.** 7w after CIA induction, mice serum was collected, IL-1a(A), IL-6(B), IL-17(C), IL-10(D), and TNF-a(E) were measured using cytokine multiplex kit. Data were analyzed using one-way ANOVA followed by Bonferroni multiple comparison test for group comparisons.  *P < 0.05, **P < 0.01, ***, p < 0.005.

**Fig. S7 Antibody levels in CIA mice intramuscularly injected with AAV6/shPD-L1.** A, Total mouse IgG level between CIA mice intramuscularly injected with AAV6/shPD-L1, AAV6/luc, and naïve mice. B, Anti-collagen II antibody level on week 3 and week 7 between CIA mice intramuscularly injected with AAV6/shPD-L1, AAV6/luc, and naïve mice. *P < 0.05, **P < 0.01, ***, p < 0.005.

**Table S1 Protein level, and the reduction percentages of antibodies and cytokines among I.V. and I.M. injected mice compared to untreated CIA mice**

|  | **Protein level at 7w (ng/mL)** | **Total IgG (%)** | **Anti-collagen II (%)** | **IL-1α** | **IL-6** | **IL-17** | **TNF- α** |
| --- | --- | --- | --- | --- | --- | --- | --- |
| I.V. | 330±160.8 | 40.5 ± 22.3 | 66.7 ± 11.6 | 50.7± 23.6 | 82.1±  13.3 | 77.5 ±13.4 | 57.2±  22.4 |
| I.M. | 182.2±48.1 | 21.7 ± 9.9 | 50 ± 17.3 | 41.4±  11.4 | 70.6±  9.1 | 74.2±  19.1 | 24.5±  12.8 |
